# Role of novel cancer gene SLITRK3 to activate NTRK3 in squamous cell lung cancer

**DOI:** 10.1186/s43556-021-00051-2

**Published:** 2021-08-30

**Authors:** Aliccia Bollig-Fischer, Bin Bao, Morenci Manning, Greg Dyson, Sharon K. Michelhaugh, Sandeep Mittal, Gerold Bepler, Hirva Mamdani

**Affiliations:** 1 Barbara Ann Karmanos Cancer Institute, Wayne State University, Detroit, MI 48201 USA; 2 Department of Oncology, Wayne State University School of Medicine, 4100 John R. St., Detroit, MI 48201 USA; 3 Department of Neurosurgery, Wayne State University School of Medicine, Detroit, MI 48201 USA; 4 Present Address: Carilion Clinic, Roanoke, VA 24104 USA; 5 Virginia Tech Fralin Biomedical Research Institute, Roanoke, Virginia 24016 USA

**Keywords:** SLITRK3, NTRK3, NTF3, Lung squamous carcinoma, Non-small cell lung cancer

## Abstract

The development of targeted therapies that inhibit cancer-driving oncogenes has improved outcomes of patients diagnosed with lung adenocarcinoma (LUAD). In contrast, patients diagnosed with lung squamous cell carcinoma (LUSC) suffer worse survival outcomes and lack effective targeted treatment options. Identification of molecular drivers of LUSC to support development of targeted treatments is urgently needed. Addressing this need, the current report introduces the novel cancer gene SLIT- and NTRK-like family member 3 (SLITRK3) and its role in activating the neurotrophic receptor tyrosine kinase 3 (NTRK3) in LUSC cells. Multiple genome-wide data sets from patient samples were produced by us or downloaded from public databases to analyze tumor gene copy number aberrations, mRNA expression and associated survival outcomes. An accompanying mechanistic study employed LUSC cell lines and multiple methods, including in situ immunofluorescence, sphere-formation assay, and fluorescence-activated cell sorting analysis of the CD133-positive cell fraction. Altogether, the results indicate that gene amplification and consequent high expression of SLITRK3 in LUSC is associated with worse outcomes and induces SLITRK3-dependent activation of NTRK3 to promote a cancer stem cell phenotype that is inhibited by existing NTRK-targeted inhibitors. Based on a recent literature search, this is the first report of a mechanistic role for SLITRK3 in cancer.

**Supplementary Information:**

The online version contains supplementary material available at 10.1186/s43556-021-00051-2.

## Introduction

Lung cancer is the leading cause of cancer-related death in the United States [1]. Non-small cell lung cancer (NSCLC) accounts for approximately 85% of all lung cancer cases, with the majority of patients presenting with incurable advanced stage disease. Recently the 5-year overall survival rate of advanced NSCLC has greatly improved due to the development of immune checkpoint inhibitors and targeted therapies that inhibit known cancer-driving oncogenes [2–4]. However, these agents preferentially benefit NSCLC patients diagnosed with lung adenocarcinoma (LUAD) [5], and current survival rates for lung squamous cell carcinoma (LUSC) remain 20–30% less than those for LUAD [6–8]. These differences in available treatments and outcomes underscore that although LUSC and LUAD tumors are each a subset of NSCLC, they are molecularly distinct diseases according to tumor genome aberration profiling [9].

Carcinogenesis and cancer progression are pathological processes fundamentally driven by aberrations in the tumor genome. Based on this understanding, much progress has been made to develop drugs that improve survival outcomes by targeting the protein products of oncogenes activated by DNA sequence mutation or by gene copy number amplification resulting in higher gene expression levels [10]. A number of targeted therapies inhibiting activated oncogenes driving LUAD have improved the survival of LUAD patients [5, 11, 12]. In contrast, LUSC tumors rarely harbor molecular alterations targeted by currently available therapies for NSCLC [5, 6], and the oncogenes driving LUSC remain unclear. Consequently, targeted treatment options are not available for the overwhelming majority of LUSC patients. Similarly, subset analyses of landmark clinical trials establishing the efficacy of immune checkpoint inhibitors in NSCLC have demonstrated greater benefit of immunotherapy in LUAD compared to LUSC [2, 3]. Therefore, identifying oncogene drivers of LUSC to support development of targeted treatments is urgently needed to improve survival outcomes of these patients. The study presented herein addresses this need.

This report introduces the novel cancer gene SLIT- and NTRK-like family member 3 (SLITRK3) and a mechanism by which it contributes to driving LUSC. SLITRK3 belongs to a family of homologous transmembrane proteins SLITRK1–6. SLITRK family members are highly expressed in the central nervous system [13]. Although they possess no intrinsic enzymatic function, they are necessary for the normal development of neurons through dimerization with neurotrophic receptor tyrosine kinase (NTRK) family members, which causes upregulation of NTRK protein levels and facilitates ligand-induced NTRK kinase activation [14]. We hypothesized a similar functional role for the aberrant expression of SLITRK3 found in LUSC. Herein, we present analyses of patient-derived tumor samples and a mechanistic study of cell lines. Results provide evidence that *SLITRK3* gene amplification frequently occurring in LUSC causes aberrant SLITRK3 overexpression and facilitates SLITRK3-dependent ligand activation of NTRK3 to induce a cancer stem cell (CSC) phenotype. Data demonstrate that LUSC cells or the environment in which LUSC tumors form are sources of the NTRK3 ligand neurotrophin growth factor NTF3 and support that currently available NTRK inhibitors may inhibit the LUSC-driving effect of SLITRK3 amplification. To our knowledge, this is the first report to recognize the recurrence of *SLITRK3* amplification and to identify a mechanistic role for SLITRK3 in cancer.

## Results

### *SLITRK3* is frequently amplified in LUSC

**Fig. 1:**
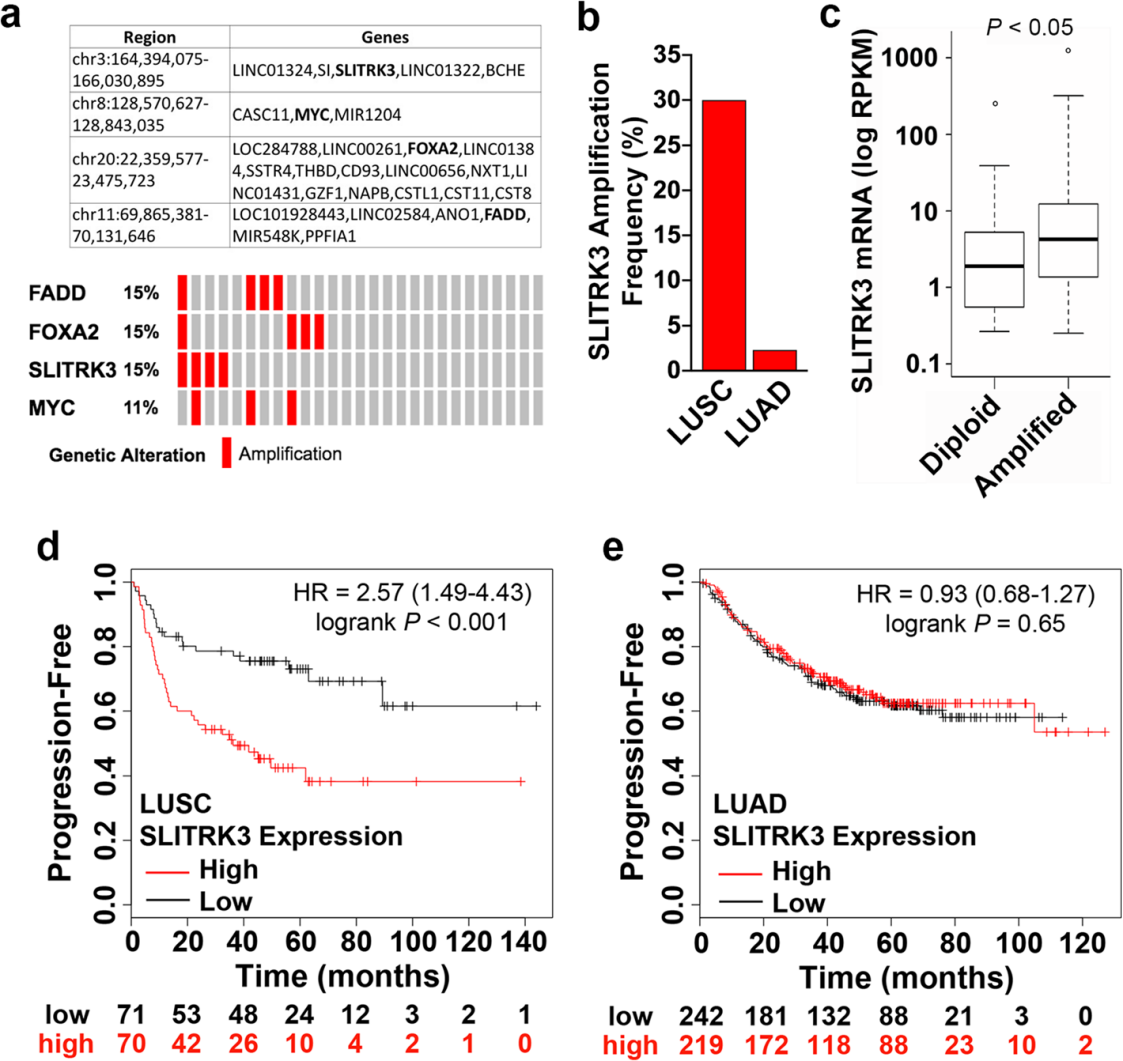
SLITRK3 amplification, overexpression and association with poor outcomes in patient data sets. **(a)** Results of GISTIC analysis of aCGH data comprising brain metastasis samples from 27 NSCLC patients identified significantly recurring focal copy number amplification of SLITRK3. **Top panel**, table listing the order of genes localized within significantly amplified regions. **Lower panel,** the frequency of events per specimen. **(b)** Frequency of SLITRK3 gene copy number amplification in LUSC and LUAD samples. **(c)** Concordance between SLITRK3 amplification and increased SLITRK3 gene expression in LUSC samples. **(d)** Kaplan-Meier plots displaying the association between high SLITRK3 mRNA expression and decreased time to first progression, specifically for LUSC. **(e)** Kaplan-Meier plot of SLITRK3 mRNA expression and decreased time to first progression data for LUAD

We first encountered SLITRK3 in exploratory analysis of 27 tissue samples from patients with advanced NSCLC using high-density array comparative genomic hybridization (aCGH). The aggregate aCGH data from these samples were analyzed using the GISTIC algorithm, which identifies significantly recurring, high-amplitude focal copy number amplification events [15]. The results included well-known cancer genes such as MYC, FOXA2 and FADD [16–18], and the unknown SLITRK3 gene (Fig. 1a). Of these 27 tissue samples that were nearly all LUAD, 4 were LUSC samples and two of these were positive for *SLITRK3* amplification. Our subsequent query of NSCLC data from The Cancer Genome Atlas (TCGA) revealed a high frequency of SLITRK3 amplification in LUSC tumors (30% versus < 5% in LUAD, Fig. 1b). Additional analysis of TCGA data identified that SLITRK3 amplification correlates with SLITRK3 mRNA overexpression (Fig. 1c). Furthermore, high-level SLITRK3 expression in tumors is associated with poor survival for LUSC patients but not for LUAD patients (Fig. 1d-e). These data are a strong indicator that frequent SLITRK3 gene amplification is a nonrandom event that plays a role in driving the biology of tumors harboring the genetic aberration, particularly LUSC tumors.

### Increased SLITRK3 expression in LUSC cells promotes the CSC phenotype, likely via NTRK3

**Fig. 2:**
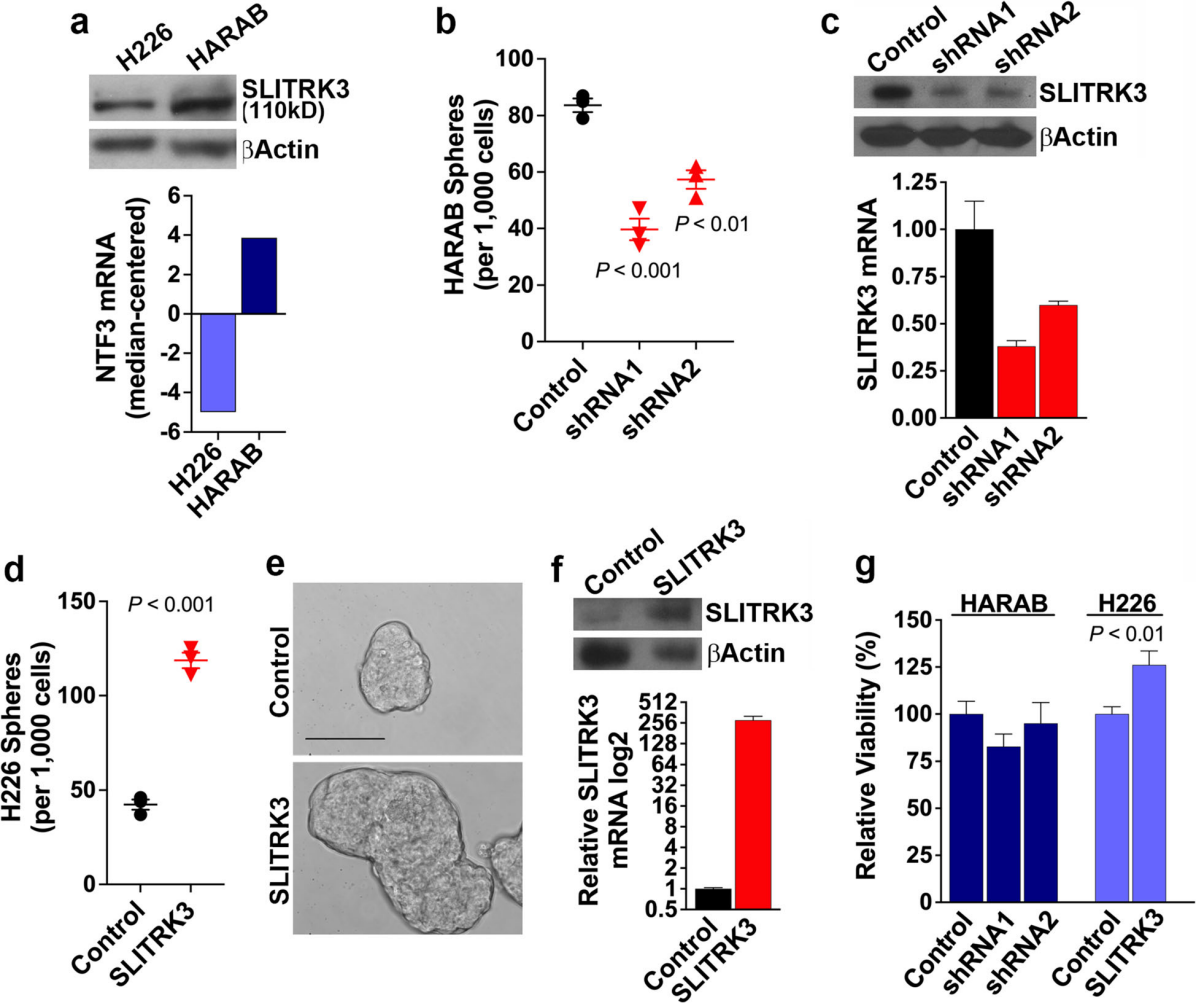
Effect on sphere-forming CSCs due to stably modifying SLITRK3 levels in LUSC cell lines. **(a**) Western blot analysis and RNA-sequencing data (normalized, log2, median-centered) displaying relative SLITRK3 levels in HARAB cells (SLITRK3-amplified) and H226 cells (SLITRK3-diploid). (**b**) Results of sphere formation assays performed with HARAB cells following stable knockdown of SLITRK3 by shRNA vectors (shRNA1 and shRNA2), relative to a nonsilencing control vector. (**c**) Western blot and qRT-PCR analyses of protein and mRNA levels, respectively, confirmed SLITRK3 knockdown in HARAB cells. **(d)** Results of sphere formation assays performed with H226 cells expressing a GFP control transgene and H226 cells stably overexpressing SLITRK3. Lines, standard error of the mean (SEM). (**e**) Representative images of spheres from GFP-expressing (control) and SLITRK3 overexpressing H226 cells. 40x magnification, Bar = 100 μm. **(f)** Western blot and qRT-PCR analyses of protein and mRNA levels, respectively, confirmed SLITRK3 overexpression in H226 cells. **(g)** Results of 3-day cell proliferation assays performed under serum-containing, attached culture conditions. HARAB cells with SLITRK3 knockdown were compared to non-silencing control cells and H226 cells overexpressing SLITRK3 were compared to GFP-expressing control cells. Bars, SEM

We moved forward to investigate whether amplification and increased expression of *SLITRK3* affects LUSC cell biology by stably modifying SLITRK3 levels in LUSC cell lines, including the H226 cell line that is *SLITRK3*-diploid, and the HARAB cell line that harbors *SLITRK3* amplification and high SLITRK3 expression (Fig. 2a). Sphere-formation assays using non-attached serum-free culture conditions revealed that relative to non-silencing control cells, stable SLITRK3 knockdown by lentiviral-transduced shRNA transgenes caused a significant decrease in HARAB sphere numbers (Fig. 2b), which represents a decrease in the fraction of viable, self-renewing CSCs in the culture [19–21]. Western blot and semi-quantitative real-time RT-PCR (qRT-PCR) analyses of protein and mRNA levels, respectively, confirmed shRNA knockdown of SLITRK3 in HARAB cells (Fig. 2c). Overexpression of SLITRK3 in H226 cells by transduction with the SLITRK3 transgene increased the number of sphere-forming CSCs and their size (Fig. 2d-e). Western blot and qRT-PCR analyses of protein and mRNA levels, respectively, confirmed SLITRK3 overexpression in H226 cells (Fig. 2f). Also, in short-term (3-day) cell proliferation assays, knockdown or overexpression of SLITRK3 in these cell lines had only modest effects (Fig. 2g), which is in line with a CSC-specific effect based on the understanding that CSCs have low proliferative potential [22]. Altogether, these data indicate that the levels of SLITRK3 expression in LUSC cells, endogenous or engineered, are concordant with the proportion of LUSC CSCs. Having linked this phenotypic effect to SLITRK3, our next goal was to uncover a relevant molecular mechanism.

As referenced in the introduction, SLITRKs are normally highly expressed in neurons where they upregulate NTRK proteins and facilitate ligand-induced NTRK kinase activation [14]. Therefore, we hypothesized that by a similar mechanism an NTRK family member is mediating the CSC-promoting effect of aberrant SLITRK3 overexpression in LUSC cells. We first needed to consider which NTRK family member was most likely involved. NTRK1, NTRK2 and NTRK3 are each a well-defined oncogene [23, 24], but NTRK3 is uniquely necessary for self-renewal of pluripotent human embryonic stem cells (ESCs) [25]. Also in ESCs, NTRK3 is transcriptionally regulated by the core stem cell factor SOX2 [25]. Recognizing that there are many parallels between ESC and CSC biology [26], we tested if what was previously reported for NTRK3 in ESCs [25] extends to LUSC CSCs.

**Fig. 3:**
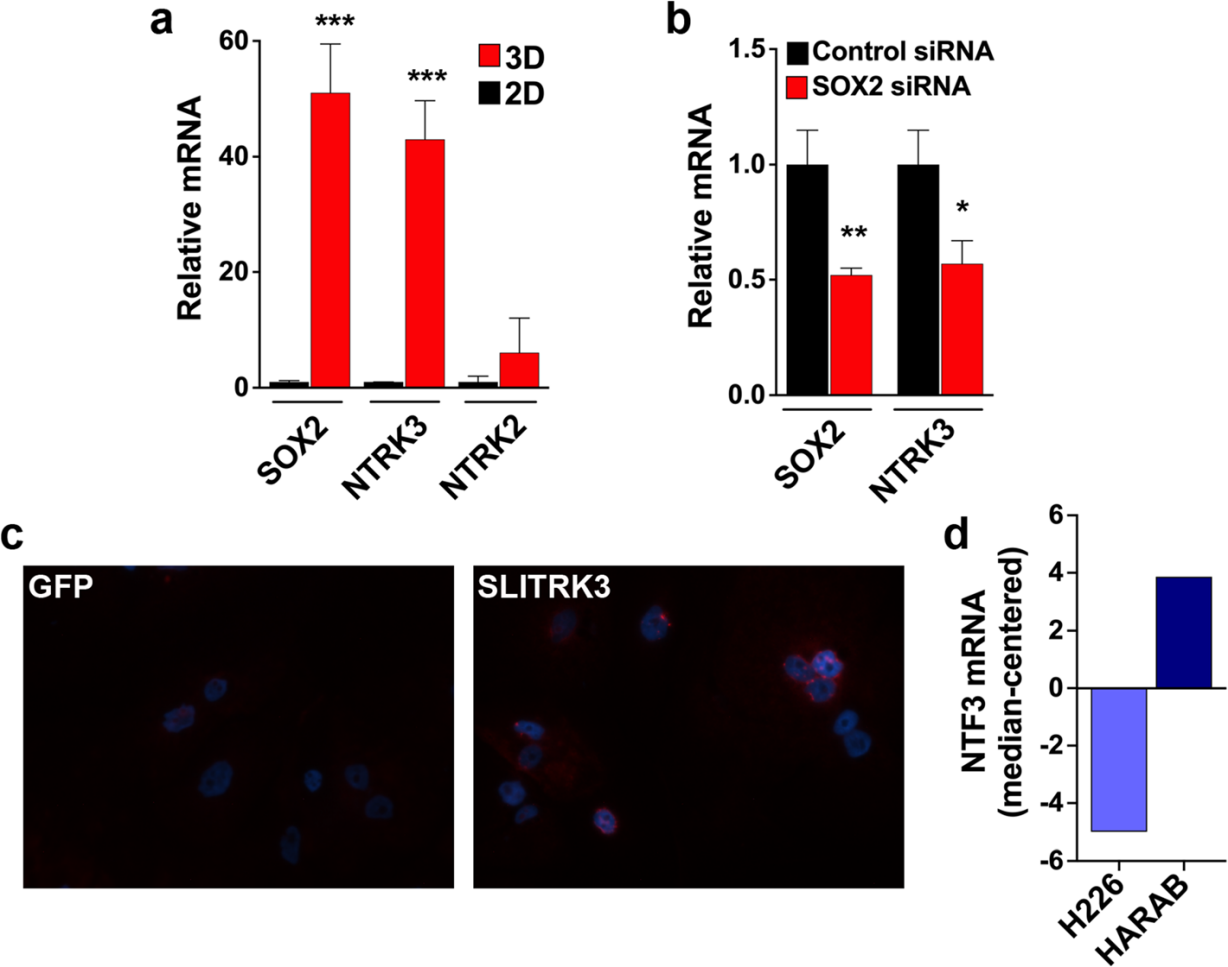
NTRK3 expression is linked to CSCs, and SLITRK3 upregulates NTRK3 protein in LUSC cells. **(a)** Expression of NTRK3 and SOX2 in H226 sphere cultures (3D) relative to levels in H226 cells cultured under attached culture conditions (2D) according to qRT-PCR analysis. Bars, SEM. **(b)** Impact of SOX2-targeted siRNA treatment on SOX2 and NTRK3 expression levels relative to non-silencing siRNA control. The ON-TARGETplus siRNA pool targeting SOX2 was applied to H226 sphere cultures and levels were measured by qRT-PCR analysis. Bars, SEM. **(c)** In situ immunofluorescent detection of NTRK3 (red) in GFP-expressing H226 cells and in H226 cells stably overexpressing SLITRK3. Nuclei (blue) were stained with DAPI. **(d)** RNA-sequencing data (FPKM, log2, median-centered) displaying relative NTF3 mRNA levels in H226 and HARAB cells. * *P* < 0.05, ** *P* < 0.01, *** *P* < 0.001

Using H226 cells, we observed that NTRK3 levels were higher in CSC-enriched sphere cultures relative to predominantly bulk cancer cells maintained in attached culture conditions (Fig. 3a). For perspective, expression levels of SOX2 were also observed to be upregulated in sphere cultures, while NTRK2 levels were low in both attached cultures and non-attached sphere growth conditions (Fig. 3a). Furthermore, knockdown of SOX2 by the transient ON-TARGETplus SMARTpool siRNA approach caused a reduction in NTRK3 transcript levels (Fig. 3b); thus, recapitulating what was previously reported for ESCs [25]. Next, we tested the prediction that SLITRK3 overexpression can cause upregulation of NTRK3 protein in LUSC cells. In situ immunofluorescent detection revealed increased NTRK3 in H226 cells stably overexpressing SLITRK3 (Fig. 3c). The punctate staining pattern is consistent with the upregulated protein being predominantly localized to endosomes in the absence of matched ligand availability, as previously reported for NTRKs [14]. The activating ligand for NTRK3 protein (also known as TRKC) is neurotrophic growth factor NTF3, and *SLITRK3*-diploid H226 cells endogenously express relatively low levels of NTF3 (Fig. 3d) corresponding with their relatively low levels of SLITRK3 (Fig. 2a). Together, these data provided the rationale for us to focus on NTRK3 and continue our study with the hypothesis that *SLITRK3*-amplification and increased SLITRK3 expression in LUSC potentiates NTF3 ligand activation of NTRK3 to induce CSCs.

### Role for SLITRK3 and the NTRK3 ligand NTF3 in the activation of NTRK3 and induction of CSCs

**Fig. 4:**
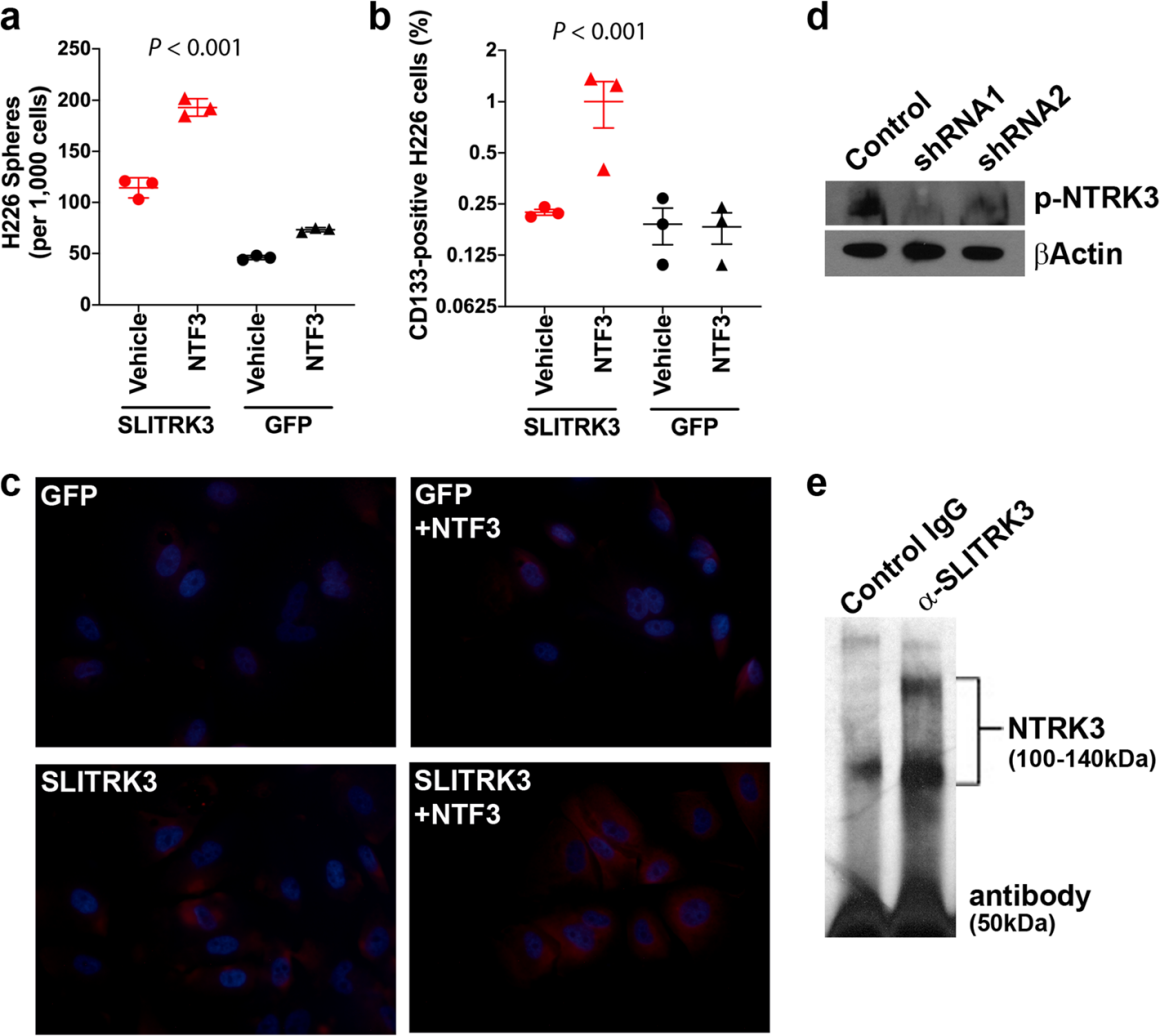
SLITRK3 overexpression potentiates the effect of NTF3 treatment to increase CSCs and NTRK3 phosphorylation. **(a)** CSC sphere formation assays assessing the effect of vehicle or 5 ng/mL NTF3 treatment (7 days) on GFP-expressing (black) or SLITRK3 overexpressing (red) H226 cells. Lines, SEM. **(b)** Impact of SLITRK3 overexpression and NTF3 treatment on the fraction of CSCs relative to total cell count based on FACS analysis of CD133+ status**.** Lines, SEM. **(c)** In situ immunofluorescent detection (red) of phosphorylated NTRK3 (p-NTRK3) in H226 cells expressing GFP or overexpressing SLITRK3, with or without NTF3 treatment. Nuclei (blue) were stained with DAPI. (**d**) Western blot analysis of p-NTRK3 levels in HARAB cells following stable knockdown of SLITRK3 by shRNA vectors (shRNA1 and shRNA2) relative to a nonsilencing control vector. (**e**) Result of immunoprecipitation performed using whole cell lysates from HARAB cells. A SLITRK3 antibody was used for immunoprecipitation and a NTRK3 antibody for Western blot detection. The bracket indicates the 2 bands that correspond to NTRK3. Rabbit IgG was used as the negative control. The band at 50 kDa corresponds to the antibody heavy chain subunit. Results are representative of 3 experiments

Next, we performed an experiment addressing whether treatment with NTF3, the NTRK3 ligand, increases the CSC fraction in the context of SLITRK3 overexpression. For this, we again used LUSC H226 cells because they express low levels of NTF3. NTF3 treatment of H226 cells stably overexpressing SLITRK3 caused a marked increase in the number of sphere-forming CSCs relative to vehicle treatment (Fig. 4a). NTF3 treatment of GFP-expressing H226 cultures elicited a relatively small increase in the number of sphere-forming CSCs (Fig. 4a), consistent with relatively low levels of SLITRK3 and an inherent expression and role for NTRK3 in the biology of LUSC CSCs.

The impact of NTF3 treatment on CSCs was also measured by fluorescence-activated cell sorting (FACS) analysis, where the fraction of CSCs was measured based on CD133-positive status. CD133 protein is a CSC-specific cell surface marker for lung cancer [27]. The FACS approach distinguishes CD133-positive CSCs from non-CSCs, also referred to as bulk cancer cells that do not express CD133. The results of FACS analyses affirmed the results above, NTF3 treatment of SLITRK3 overexpressing H226 cells markedly increased the proportion of CSCs in the culture (Fig. 4b). NTF3 treatment of GFP-expressing cells appeared to have little effect on the proportion of CSCs (Fig. 4b**).** The raw FACS data are in Supplementary Fig. 1.

In situ immunofluorescent detection of phosphorylated NTRK3, a key measure of the activated receptor tyrosine kinase, yielded concordant results. NTF3 treatment of GFP-expressing H226 control cells appears to have had a small effect (Fig. 4c); but unequivocally, NTF3 treatment of SLITRK3 overexpressing H226 cells induced the highest levels of phosphorylated NTRK3 (Fig. 4c). The staining pattern of phosphorylated NTRK3 concurs with reported cytoplasmic distribution and membrane-localization of NTRKs [14], particularly with added ligand. The levels of phosphorylated NTRK3 were increased in H226 cells stably overexpressing SLITRK3 relative to GFP-expressing controls (Fig. 4c). This induction is consistent with the presence of some endogenous NTF3 expression in the cultures (Fig. 3e) and the induction of CSCs due to SLITRK3 overexpression alone (Fig. 2d).

We confirmed that modulating SLITRK3 levels affects phosphorylated NTRK3 levels using the HARAB cell line, which expresses relatively high levels of SLITRK3 and NTF3. Compared to non-silencing control cells, stable SLITRK3 knockdown by lentiviral-transduced shRNA transgenes caused a decrease in phosphorylated NTRK3 levels (Fig. 4d). To determine whether SLITRK3 and NTRK3 are physically associated in LUSC cells, we performed immunoprecipitation assays using whole cell lysates from HARAB cells, a SLITRK3 antibody for immunoprecipitating and a NTRK3 antibody for Western blot detection. The results demonstrated that SLITRK3 and NTRK3 proteins interact (Fig. 4e).

**Fig. 5:**
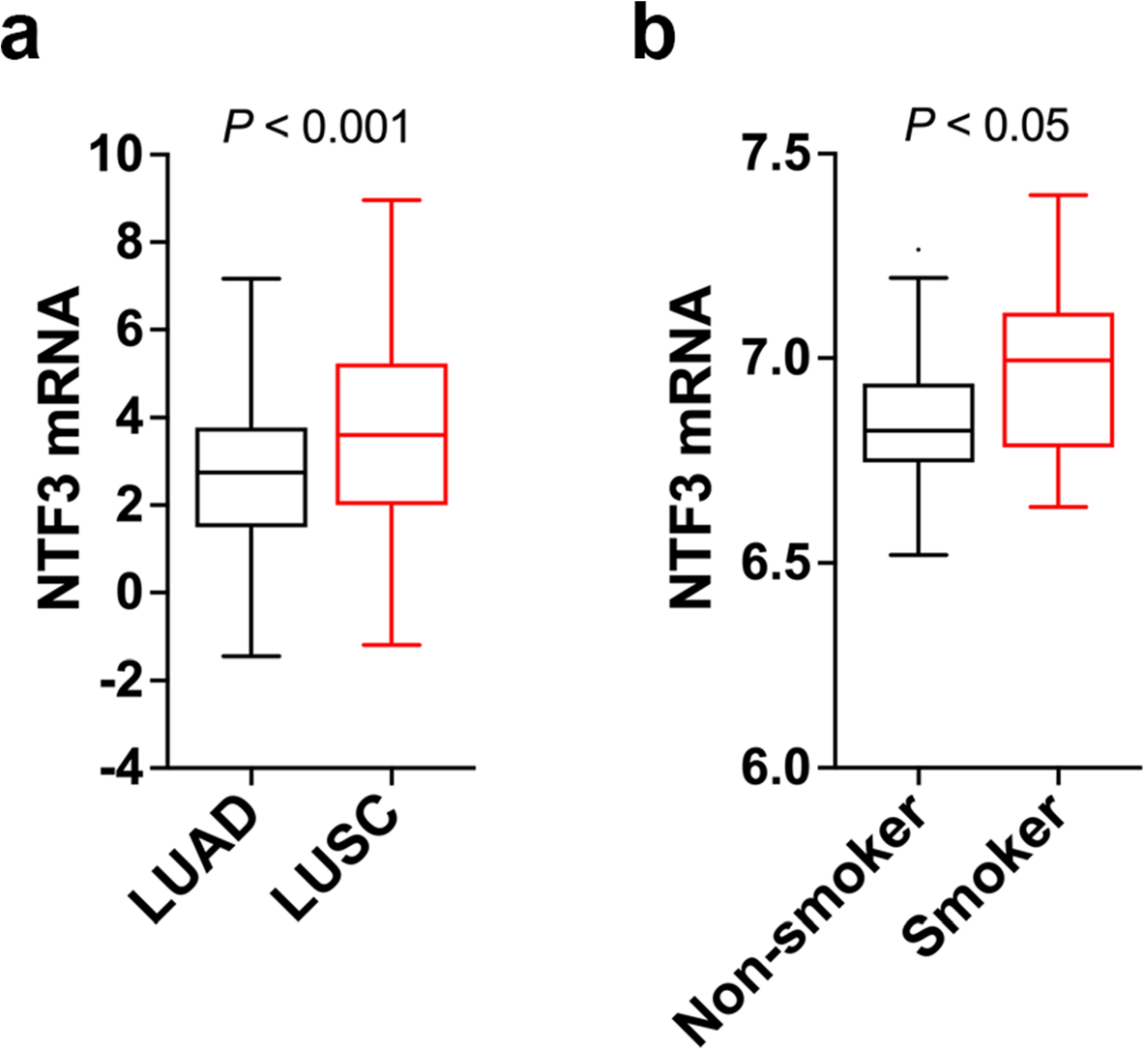
NTF3 levels are increased in LUSC tumors and bronchial airways of smokers. **(a)** Tumor NTF3 levels, LUAD versus LUSC. The dataset is normalized, log2 RNA-sequencing data from TCGA. Number (n) of patient specimens per group: 250 LUAD, 284 LUSC. **(b)** NTF3 levels in a non-cancer cohort comparing bronchial brushings from smokers (n = 16) and healthy controls (n = 28). The dataset is normalized, log2 gene expression microarray data from GEO accession number GSE4302

The results from cell line analyses underscore a role for NTF3 in SLITRK3-dependent activation of NTRK3 and suggest that the source of NTF3 expression may be LUSC cells or the tissue environment where LUSC arises. We next examined if analysis of relevant human tissue samples supports this idea. According to TCGA mRNA data, NTF3 is higher in LUSC tumors relative to LUAD (Fig. 5a). In addition, analysis of mRNA data derived from the airway brushings of a noncancer cohort revealed that NTF3 is higher in the bronchial airway epithelium of current cigarette smokers relative to nonsmoker controls (Fig. 5b). This result aligns with current cigarette smoking being a major risk factor for the development of LUSC and that LUSC forms from cells lining bronchial airways [28–30].

**Fig. 6:**
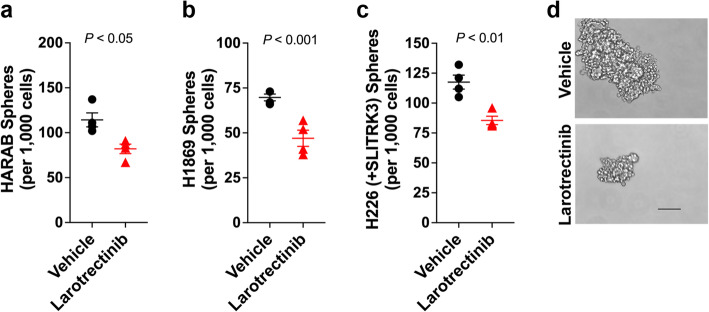
A pan-NTRK SMKI inhibits CSCs. CSC sphere formation assays were performed on **(a-b)** HARAB and H1869 LUSC cell lines, each with endogenous SLITRK3 amplification, and **(c)** H226 cells transduced to overexpress SLITRK3. Cultures were treated with NTRK inhibitor larotrectinib (1 μM) or vehicle. Lines, SEM. **(d)** Representative images of HARAB spheres, vehicle control and larotrectinib-treated (7 days, 1 μM). 20x magnification, Bar = 100 μm

Extrapolating from the whole of the evidence presented above, we postulated that an existing NTRK-targeted small molecule kinase inhibitor (SMKI) drug should have a demonstrable inhibitory effect on *SLITRK3*-amplified LUSC cells with their relatively high proportion of CSCs. Accordingly, treatment of HARAB cultures with larotrectinib, a pan-NTRK SMKI (there are no SMKIs specific to an individual NTRK family member), significantly reduced the numbers of sphere-forming CSCs in 7-day sphere formation assays (Fig. 6a). We extended this experiment to include the LUSC cell line NIH-1869, which also has *SLITRK3* amplification, and observed a similar anti-CSC effect with larotrectinib treatment (Fig. 6b). The drug treatment also counteracted sphere-forming CSCs induced by stable SLITRK3 overexpression in H226 cells (Fig. 6c). Furthermore, the treatment of HARAB cells, which form particularly large spheres in the 7-day assay, demonstrated that larotrectinib markedly reduced the outgrowth potential of the spheres that did form (Fig. 6d).

## Discussion

The outcomes of our patient specimen data analysis and mechanistic study of LUSC cell lines provide reinforcing lines of evidence to support that copy number amplification of *SLITRK3* is not a random passenger aberration in the LUSC tumor genome but has a role in activating NTRK3 and thereby promoting CSCs. CSCs are the recognized source of primary malignant tumor initiation and they give rise to metastases and tumor recurrence [22, 31, 32]. The biological effect of *SLITRK3* amplification to increase the proportion of CSCs is consistent with shorter time to progression associated with high SLITRK3 expression in patient tumors (Fig. 1D). The results of our mechanistic study also bore out the prediction that *SLITRK3* amplification and aberrant expression of SLITRK3 in LUSC cells serves a role similar to the role of SLITRK family members in neurons where they are normally highly expressed [13]. They dimerize with neurotrophic receptor tyrosine kinase (NTRK) family members, which causes upregulation of NTRK protein levels and facilitates ligand-induced NTRK kinase activation [14]. Furthermore, *SLITRK3* amplification recurring in LUSC at a frequency of 30% with the mechanistic role we have uncovered can explain why a previous tumor immunostaining study reported NTRK3 protein is absent in LUAD samples but detected in approximately 30% of LUSC samples [33]. We plan to extend these findings in future work using LUSC patient tumor samples to examine if NTRK3 detection correlates with SLITRK3 amplification and high levels of lung CSC markers, such as CD133 or ALDH1 [34].

NTRK family members, including NTRK3, are well-established oncogenes targeted by FDA-approved SMKI drugs [35]. Thus far these agents have only been explored to treat tumors harboring NTRK fusion genes occurring at < 1% frequency in NSCLC [36]. These fusion genes lack the ligand-binding domain, and the chimeric protein product is constitutively membrane-localized and kinase-activated [36]. Our findings indicate that NTRK3 activation dependent on aberrant SLITRK3 expression due to *SLITRK3* amplification in LUSC may also be targeted by currently available NTRK inhibitors. Therefore, this alternative mechanism to activate NTRK3 involving 30% of LUSC with *SLITRK3* amplification is substantial and potentially clinically meaningful and justifies further pre-clinical and clinical research.

It appears to be relevant that the focally amplified *SLITRK3* gene originates from within the boundaries of the previously recognized 3q26-q29 region of broad, low-level copy number gain that is estimated to occur in 85% of LUSC and also harbors the SOX2 gene [37]. In our own lung metastatic brain tumor sample set, SOX2 was not among genes focally amplified according to GISTIC analysis, but tumors with SLITRK3 amplification displayed SOX2 copy number gains (data available at GSE157515). SOX2 copy number gains yield increased SOX2 expression, which has received a great deal of attention in the literature [38]. The evidence presented herein suggests that a novel cooperative relationship exists: while SOX2 transcriptionally upregulates NTRK3 mRNA expression (reference [25] and in Fig. 3b), SLITRK3 amplification and/or overexpression and NTF3 availability are essential to achieve fully enhanced NTRK3 protein-level activation.

Abundant NTF3 can originate from LUSC cells (Fig. 3**,** Fig. 5a) or from the tissue environment at the site of origin of LUSC tumors. It is almost certainly not coincidental that NTF3 levels are high in bronchial airway tissue of current smokers who are at high risk of developing LUSC relative to nonsmokers (Fig. 5b), which likely reflects that the bronchial airways of current smokers are infiltrated by white blood cells that express and release NTF3 into the extracellular tissue environment [39–41]. These data and interpretations are also consistent with LUSC forming from squamous cells lining proximal bronchial airways and that the incidence of LUSC is more strongly associated with smoking than LUAD is [28]. Altogether, the evidence supports the hypothesis that NTF3 plays a critical role in driving LUSC. Moreover, the mechanism that we discovered is consistent with the observation that genome aberrations alone may not drive cancer [42], adding that when a genomic aberration does drive cancer, it is in part due to a selective advantage conferred by the host tissue environment.

## Conclusion

Multiple lines of evidence presented herein converge on the novel findings that *SLITRK3* gene amplification frequently recurring in LUSC is associated with upregulated SLITRK3 expression, which results in SLITRK3-dependent activation of NTRK3. High level of NTF3 in the bronchial airways may play a role in full enhancement of SLITRK3 driven NTRK3 activation. This warrants further study to realize how SLITRK3-dependent NTRK3 activation in LUSC may translate to expanding the use of currently available, FDA-approved NTRK inhibitors to a larger patient population that carries a worse prognosis.

## Materials and methods

### Array comparative genomic hybridization (aCGH) analysis of tumor specimens

De-identified, fresh-frozen, surgically resected brain metastases from 27 NSCLC patients treated at Karmanos Cancer Institute were collected and analyzed. The analysis was performed at the Clinical Genomic Laboratory at Karmanos Cancer Institute. The work was pre-approved by the Wayne State University Institutional Review Board. Written informed consent was obtained from all donating patients and methods conformed to the standards set by the Declaration of Helsinki. Genomic DNA was isolated using the EZ1 Advanced workstation and DNA Tissue Kit (Qiagen, Germantown, MD). A Trinean DropSense96 Spectrophotometer (PerkinElmer, Waltham, MA) and TapeStation (Agilent, Santa Clara, CA) were used to measure DNA quantity and quality. aCGH analysis employed the Agilent SureScan platform and the Cancer Cytogenomic CGH + SNP (4 × 180 k) array following manufacturer’s recommendations. Agilent’s SNP-characterized normal human DNA (male product no. 5190–3796 or female product no. 5190–3797, used according to patient gender) was the reference sample. Probe fluorescence intensity data was extracted from scanned microarray images using Agilent Cytogenomics software. GC Correction, Diploid Peak Centralization (normalization) and Aberration Detection Method 2 (quality control) algorithms were also applied using this software. All extracted (ratio) data, copy number variation intervals and genes mapping to intervals were assembled in files available at Gene Expression Omnibus (GEO) accession number GSE157515.

### Bioinformatic and statistical analysis

The GISTIC [15] algorithm was applied to aggregate aCGH data from 27 metastatic tumors using NEXUS Copy Number software (Biodiscovery, El Segundo, CA). SLITRK3 copy number analysis data and gene expression RNA sequencing data from The Cancer Genome Atlas (TCGA) [43] were downloaded via cBioPortal [44]. RSEM (RNA-Seq by Expectation-Maximization) normalized RNA sequencing data for NTF3 gene expression in LUSC and LUAD were from TCGA accessed via FireBrowse.org. Bronchial airway NTF3 gene expression microarray data from smokers and nonsmokers in a noncancer cohort, RMA (robust multichip average) normalized, were acquired from GEO accession number GSE4302. Cell line DNA copy number and FPKM (fragments per kilobase of exon model per million reads mapped) normalized RNA-sequencing data were acquired from the Cell Line Encyclopedia (broadinstitute.org/ccle).

All experiments were performed multiple times, yielding data for analysis from at least 3 biologically independent samples. Linear regression or Student’s t-test (two-sided) were applied to compare the means when two conditions were compared. Pearson’s test was used for analysis of correlation. For analysis of 3 or more groups, a mixed-model approach was applied. These analyses were performed using Bioconductor R 3.3.2. Combined datasets GSE29013, GSE50081 and GSE8894 were analyzed by log rank test with KM Plotter [45], using Jetset [46] selected Affymetrix probe 206732_at and time to first progression as the metric. *P* values ≤0.05 are reported as significant and *P ≤ 0.05, **P < 0.01, ***P < 0.001 indicates level of significance.

### Cell culture, treatments and stable gene overexpression and knockdown

NCI-H226 and NCI-H1869 cell lines were acquired from ATCC (Manassas, VA) and HARAB from JCRB Cell Bank (Osaka, Japan) for this study. They were maintained according to suppliers’ instruction in RPMI1640 media containing 5–10% FBS. For each frozen aliquot, experiments were performed after at least 2 and fewer than 12 passages. Lentivirus-mediated, shRNA knockdown of SLITRK3 expression was done as previously described by us using the Open Biosystems Expression Arrest GIPZ lentiviral shRNAmir system (vector ID no. V3LHS332342 for shRNA1 and V2LHS96099 for shRNA2). Lentiviral particles from Cyagen Biosciences (Santa Clara, CA) were used according to supplier recommendations to stably transduce vectors expressing human SLITRK3 (NM_001752.3) or GFP. Expression of the transgene and shRNA were under control of a CMV promoter. These same vectors also expressed a puromycin selection gene, utilized for stable selection. SOX2 knockdown employed ON-TARGET plus SMART pool siRNA chemically modified to disrupt interaction with transcripts containing only partial complementarity, thereby disrupting potential off-target effects (Dharmacon, Lafayette, CO, product no. L-011778-00-0005). NTF3 used in cell culture at 5 or 50 ng/ml concentration, as indicated in figure legends, was from Sigma-Aldrich (St. Louis, MO; product no. SRP3128). Larotrectinib, used at 1 μM, was from Selleckchem (Houston, TX; product no. S7960).

### Semi-quantitative real-time RT-PCR (qRT-PCR) analysis

RNA was isolated from cultured cell lines using the Qiagen RNeasy kit (Valencia, CA). RNA was converted to cDNA using the High-Capacity RNA-to-cDNA kit (Thermo Fisher, Waltham, MA). 100 ng cDNA was combined with SYBR Green PCR Master Mix (Thermo Fisher) in 20 μL reactions in 96-well plates. Reactions were run in triplicate using the StepOnePlus Real-Time PCR System (Thermo Fisher). Relative levels of expression were calculated using the delta-delta Ct method [47]. Primer pairs included: SLITRK3 (Primerbank [48] ID: 40217819c1), NTRK3 (Primerbank ID: 340745350c1), NTRK2 (Primerbank ID: 65506645c1), SOX2 (Primerbank ID: 325651854c2); and NANOG Primerbank ID: 153945815c3. βActin (forward: CCCAGCACAATGAAGATCAA, reverse: ACATCTGCTGGAAGGTGGAC) served as the loading control gene.

### Western blot analysis and immunoprecipitation assay

Protein was isolated from cell cultures using RIPA buffer (Thermo Fisher) and concentration measured using the Bradford Assay (Bio-Rad, Hercules, CA) following manufacturer’s instruction. For Western blot analysis, 50–100 μg protein was separated on a 10% SDS-PAGE gel and transferred to a nitrocellulose membrane (GE Healthcare Life Sciences, Pittsburgh, PA) that was then blocked with 5% non-fat dried milk in 1× tris-buffered saline plus 0.1% Tween-20 and probed with primary antibodies and secondary horseradish peroxidase-conjugated antibodies following supplier’s recommendations. Primary antibodies included: NTRK3 (product no. 3376S, Cell Signaling, Danvers, MA), phosphorylated NTRK3 (Novus Biologicals, Littleton, CO, product no. NBP1–03448), and anti-SLITRK3 (GeneTex, Irvine, CA, product no. GTX85417 and Proteintech, Rosemont, IL, product no. 21649–1-AP). Also, anti-βActin (Sigma-Aldrich product no. A3853) served as the loading control. Protein bands were visualized using Pierce ECL Western Blotting Substrate (Thermo Fisher) and autoradiography film (Denville Scientific, Holliston, MA). Following manufacturer’s instruction, the Pierce Classic IP column kit (Thermo Fisher) was used for the immunoprecipitation assay. Briefly, 10 μg of SLITRK3 antibody (Proteintech) or rabbit IgG control (Thermo Fisher) was combined with 1 mg of whole cell protein lysate from HARAB cells (pre-cleared with control agarose resin) and incubated at 4 °C overnight with constant rotation. Immune complexes were bound to protein A/G agarose beads, rotating for 1 h at room temperature. The beads were then washed three times. Beads were suspended in sample elution buffer and heated to 100 °C before the final column centrifugation and collection step. Subsequent Western blot analysis was performed using the total sample eluent and NTRK3 antibody.

### In situ immunofluorescence analysis

In situ immunofluorescence analysis was performed on H226 cells transduced with SLITRK3 or GFP that were plated on glass cover slips and cultured with or without NTF3. Cells were washed three times with PBS, fixed for 15 min in 5% formaldehyde in PBS solution, rehydrated by three washes with PBS, pre-incubated 10 min in 2% BSA-PBS solution, and incubated 45 min at room temperature with primary antibody against NTRK3 (product no. 3376S; Cell Signaling. Danvers, MA) or phosphorylated NTRK3 (Novus Biologicals, Littleton, CO; product no. NBP1–03448) diluted 1:200 in 2% BSA-PBS solution. Bound antibody was detected by staining with a secondary goat anti-rabbit Alexa Fluor 594-conjugated antibody (Invitrogen, Carlsbad, CA) diluted 1:500 in 2% BSA-PBS solution, and incubated for 45 min at room temperature, followed by 3 times of washing with 0.05% Tween-20/PBS solution. Cover slips were fixed to slides with a drop of ProLong Gold Anti-fade reagent containing 4′,6-diamidino-2-phenylindole (DAPI) to stain nuclei (Invitrogen). Images were recorded at 40× magnification using an EVOS Digital Inverted Microscope (Advanced Microscopy Group, Mill Creek, WA).

### Cell proliferation assay

Following the manufacturer’s instructions, the CellTiter-Glo luminescent assay kit (Promega, Madison, WI) was used to measure the amount of ATP, which is proportional to the number of viable cells in attached cultures. Briefly, 3000 cells were seeded per well in 100 μl of standard serum-containing culture media in 96-well plates and measurements were taken after 72 h using a Synergy 2 multi-mode microplate reader (BioTek Instruments, Winooski, VT).

### Sphere-formation assay and fluorescence-activated cell sorting (FACS) analysis

The presence and viability of CSCs was examined in cancer cell line cultures using the sphere-formation assay. 1000 single cells were seeded in 6-well ultra-low attachment plates (Corning Inc., Corning, NY) with serum-free sphere formation medium comprising 1:1 DMEM:F-12 media plus with B-27 and N-2 supplements (Thermo Fisher) maintained at 37 °C, 5% CO_2_. After 7 days of incubation, the spheres formed were counted and reported as a fraction of the total number of cells seeded. Images were acquired using a Nikon Eclipse TE2000-U inverted microscope. The total number of lung cancer cells and cells expressing CSC-specific marker CD133 in each sample were counted by FACS analysis using the BD LSR II Flow Cytometer (BD Biosciences, San Jose, CA) and a fluorochrome-labeled monoclonal antibody against human CD133 from Miltenyi Biotec (Cologne, Germany, catalogue number 130–090-854).

## Supplementary Information

**Additional file 1: Supplementary Fig. 1.** Density plots from FACS analysis of CD133-positive cell fractions compiled from 3 independent biological replicates for each of 4 groups: H226 cell line transduced with GFP plus and minus NTF3, and H226 cell line transduced with SLITRK3 plus and minus NTF3. Non-staining control data panels for each transduced cell line are included.

## Data Availability

aCGH data are available at Gene Expression Omnibus accession GSE157515.

## Abbreviations

aCGH: array comparative genomic hybridization
CSC: cancer stem cell
ESC: embryonic stem cell
FACS: fluorescence-activated cell sorting
FDA: U.S. Food and Drug Administration
GFP: green fluorescent protein
GISTIC: Genomic Identification of Significant Targets in Cancer
LUAD: lung adenocarcinoma
LUSC: lung squamous cell carcinoma
NSCLC: non-small cell lung cancer
NTF3: neurotrophin 3
NTRK3: neurotrophic receptor tyrosine kinase 3
SLITRK3: SLIT- and NTRK-like family member 3
TCGA: The Cancer Genome Atlas
