# Supplementary material for: Role of novel cancer gene SLITRK3 to activate NTRK3 in squamous cell lung cancer
Source: Mol Biomed. 2021 Aug 30;2:26. doi: 10.1186/s43556-021-00051-2 (PMC8607376; doi:10.1186/s43556-021-00051-2)
Supplement: Supplementary file 1 — Additional file 1: Supplementary Fig. 1. Density plots from FACS analysis of CD133-positive cell fractions compiled from 3 independent biological replicates for each of 4 groups: H226 cell line transduced with GFP plus and minus NTF3, and H226 cell line transduced with SLITRK3 plus and minus NTF3. Non-staining control data panels for each transduced cell line are included. [file 43556_2021_51_MOESM1_ESM.pdf]

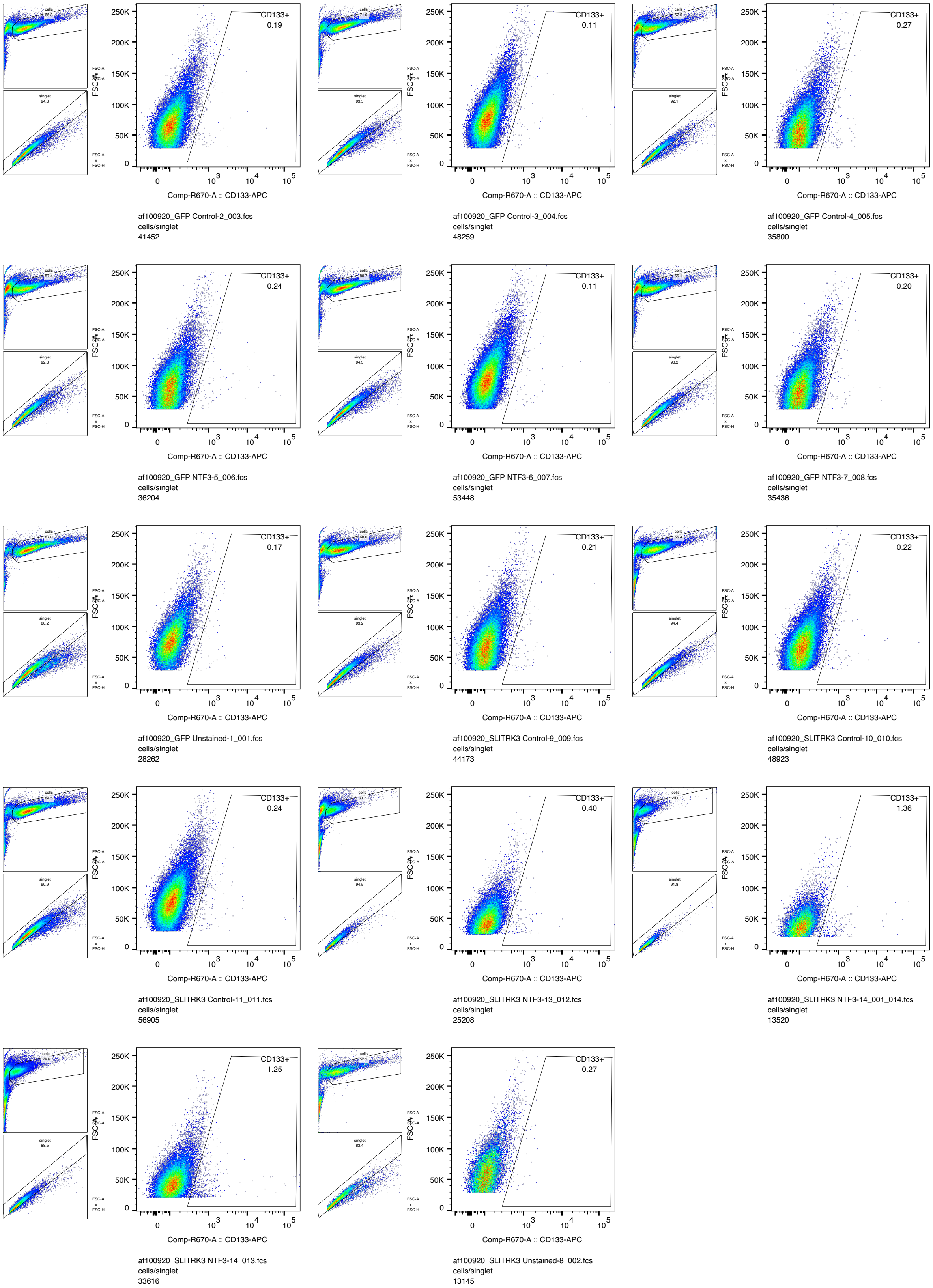

Supplementary Fig. 1. Density plots from FACS analysis of CD133-positive cell fractions compiled from 3 independent biological replicates for each of 4 groups: H226 cells transduced with GFP plus and minus NTF3, and H226 cells transduced with SLITRK3 plus and minus NTF3. Non-staining control data panels for each transduced cell line are included.
